# Pediatric acute kidney injury and adverse health outcomes: using a foundational framework to evaluate a causal link

**DOI:** 10.1007/s00467-024-06437-y

**Published:** 2024-07-01

**Authors:** Catherine Morgan, Emma Forest, Emma Ulrich, Scott Sutherland

**Affiliations:** 1 Department of Pediatrics, Division of Nephrology, University of Alberta, Edmonton, AB Canada; 2 School of Public Health, University of Alberta, Edmonton, AB Canada; 3 Department of Pediatrics, Division of Nephrology, Center for Academic Medicine, Stanford University School of Medicine, Palo Alto, CA USA

**Keywords:** Acute kidney injury, Causality, Pediatric, Bradford Hill, Outcomes

## Abstract

Acute kidney injury (AKI) is a major global health problem, expensive to manage, and its associations with negative pediatric health outcomes have been clearly demonstrated. One of the most fundamental questions to consider as we use previous epidemiological information to advance research and care paradigms is the strength of the causal link between pediatric AKI and health outcomes. In this review, we apply the foundational framework of the Bradford Hill criteria to evaluate the extent to which a causal link exists between AKI and the associated adverse outcomes in children. Available data in children support a causal link between AKI and short-term outcomes including mortality, length of stay, and ventilation time. Clarifying the causal nature of longer term associations requires further high-quality observational studies in children, careful consideration of what defines the most meaningful and measurable longer term outcomes after pediatric AKI, and integration of evolving biological data related to mechanisms of disease. Preventing or mitigating AKI should lead to improved outcomes. Demonstrating such reversibility will solidify confidence in the causal relationship, improve child health, and highlight an aspect which is highly relevant to clinicians, scientists, and policy makers.

**Graphical abstract:**

**Figure:**
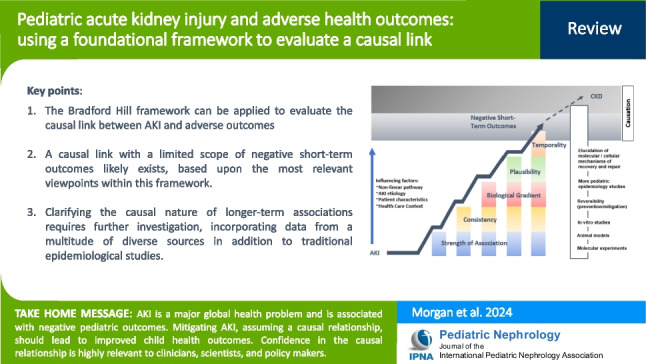
A higher resolution version of the Graphical abstract is available as Supplementary information

**Supplementary Information:**

The online version contains supplementary material available at 10.1007/s00467-024-06437-y.

## Introduction

Progress in pediatric acute kidney injury (AKI) knowledge generation has been vast over the last two decades, including expanding epidemiology, understanding AKI pathophysiology and its long-lasting consequences, as well as risk prediction in children and novel biomarker use [1–10]. This progress has set a solid foundation on which to build future work, with the goal of improving global child AKI health outcomes [11].

Underpinning many of the current priorities identified for pediatric AKI research, education, practice, and advocacy [11] is the hypothesis that a direct or indirect causal relationship exists between AKI events and AKI related sequelae. It is the hope that preventing or mitigating the severity of AKI will improve outcomes that drives interventional and quality improvement strategies. One of the most fundamental questions to consider as we use previous epidemiological information to advance research and care paradigms is the strength of this causal link between pediatric AKI and previously evaluated health outcomes. Although individual studies may suggest a causal link between pediatric AKI and a limited scope of short-term outcomes [12, 13], multidisciplinary experts in this field agree that associations demonstrated in one study cannot imply causality, and a broader view synthesizing multiple sources of information is required to bridge pediatric AKI knowledge gaps, including the causal relationship with outcomes [10].

**Fig. 1:**
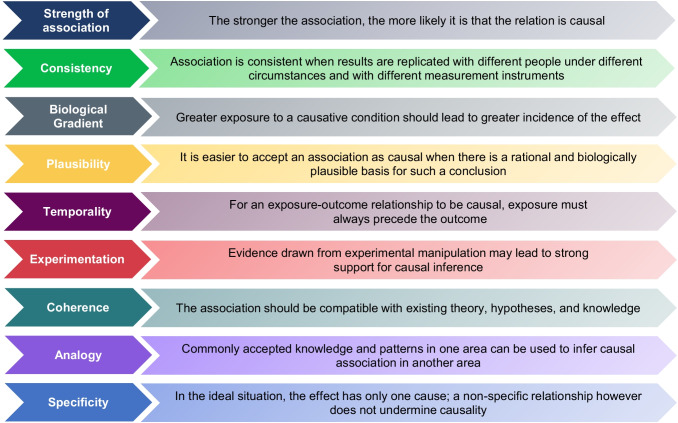
Bradford Hill’s considerations for causality inference in observational associations

As with other epidemiological data, causal assessment mechanisms can be applied to the current body of pediatric AKI literature to interrogate the relationship between events and outcomes. One of the fundamental mechanisms to achieve this is the Bradford Hill criteria. These criteria, which have served as a foundation for causal assessment since their initial publication in 1965 [14], consider nine specific viewpoints (Fig. 1): plausibility, temporality, strength of association, specificity, analogy, experimentation, coherence, biological gradient, and consistency. In the assessment of underlying causal relationships in epidemiologic evidence, these criteria have been favorably compared with more modern approaches such as directed acyclical graphs, sufficient-component cause models, and the GRADE methodology (grading of recommendations, assessment, development, and evaluation), with significant overlap across the methods [15]. This underscores the enduring importance of the Bradford Hill criteria and is consistent with the available literature which demonstrates that these nine viewpoints account for the most relevant causal considerations [15, 16].

The following review applies the foundational framework of the Bradford Hill criteria to evaluate the extent to which a causal link exists between AKI and the associated adverse outcomes in children (see summary Tables 1 and 2). The analysis will focus on strength of association, consistency, biological gradient, plausibility, temporality, and experimentation as evidence suggests that the viewpoints of coherence, analogy, and specificity may have limited usefulness in modern causal assessment [15]. In addition, given how widely used the GRADE approach is in assessing quality of evidence and grading strength of healthcare recommendations, and is an approach that has adopted most of Bradford Hill’s criteria [17], we have considered these Bradford Hill viewpoints within the GRADE framework (see Tables 1 and 2).

**Table 1:** Bradford Hill’s considerations for pediatric AKI–negative short-term outcome causality inference

Criteria	Summary of support for causal link	Pediatric supporting evidence	Additional consideration by applying GRADE methodology [17]^a^
Strength of association and consistency	**Robust supporting evidence:** • Studies indicate a strong association between AKI and short-term outcomes • Multiple epidemiological studies show a consistent association despite varying populations, epidemiological methods, and locations	Schneider (2010), Alkandari (2011), Askenazi (2011), Li (2011), Binder (2012), Selewski (2013), Jetton (2017), Kaddourah (2017), Alobaidi (2020) Schneider (2010), Alkandari (2011), Askenazi (2011), Li (2011), Binder (2012), Selewski (2013), Sutherland (2015), Mehta (2016), Jetton (2017), Kaddourah (2017), Hsu (2018), Alobaidi (2020), Searns (2020), Sarkar (2021)	Evidence from NRSs estimate a large effect and therefore upgraded on the basis that confounding is less likely to entirely remove the observed association Within similar populations, there is minimal inconsistency in effect sizes, increasing confidence about the effect of the exposure on the outcome
	**Limitations:** • Not all AKI is associated with poorer outcomes; underlying risk for outcomes likely varies across populations • Factors that may confound the relationship are difficult to eliminate, although can be appropriately controlled for	Sutherland (2015)	
Biological gradient	**Robust supporting evidence:** • Data support the presence of a biological gradient between the severity of AKI and the magnitude of short- term negative outcomes	Sanchez-Pinto (2015), Jetton (2017), Kaddourah (2017), Sutherland (2021)	A dose–response gradient is present and therefore, evidence upgraded because, alongside a strong effect, it indicates that the effect is less likely due to residual confounding
Plausibility	**Biologically plausible:** • AKI sequelae are accepted mechanisms of mortality/ morbidity; large proportion of negative outcome risk attributable in statistical modeling to metabolic and fluid related complications • Clinical and experimental findings illuminate additional complex but plausible exposure-to-effect paradigms	White (2012), Libório (2015), Lee (2018), Leaf (2019), Pickkers (2021)	GRADE upgrades for adjustment for plausible confounding, but not plausibility of relationship; most recent large, high-quality studies have adjusted for plausible confounding
Temporality	**Supportive:** • Temporal sequence supports causation • Biologically plausible mechanism linking AKI and outcome fits within the time course	Jetton (2017), Kaddourah (2017), Alobaidi (2021)	Evidence starts as lower quality as no RCTs, which by default establish a temporal relationship There are now many cohort studies (some prospective) including concurrent control groups which increases the quality of the evidence as more likely to ensure that AKI precedes the outcome
Experimentation	**Indeterminate** • Some evidence that mitigating established AKI sequelae may change outcomes • Surrogate information that preventing AKI could modify outcomes	Kwiatkowski (2015), Ryerson (2015), Goldstein (2023), Ulrich (2024)	Evidence is from NRSs and therefore downgraded as GRADE privileges effect estimates from randomized studies
	**Limitations** • Ongoing generation of evidence from experimental manipulation of AKI and its complications is required		

*AKI* acute kidney injury; *GRADE* grading of recommendations, assessment, development, and evaluation); *NRS* non randomized study; *RCT* randomized controlled trial

^a^GRADE defines the quality of evidence as the confidence in an estimate of effect (causal relation) from a body of evidence; therefore, the term upgrading is used when this confidence is increased and the term downgrading when this confidence is lowered

**Table 2:** Bradford Hill’s considerations for pediatric AKI–CKD causality inference

Criteria	Summary of support for causal link	Pediatric supporting evidence	Additional consideration by applying GRADE methodology [17]^a^
Strength of association and consistency	**Indeterminate** • In the general PICU population, AKI is associated with CKD • In the cardiac surgery population, the association between AKI and long-term outcomes is less clear	Menon (2014), Cooper (2016), Hollander (2016), Al-Otaibi (2017), Greenberg (2018), Hessey (2019), Benisty (2020), Hessey (2020), Huynh (2020), Menon (2020), Zappitell (2020), Hessey (2021)	Evidence from NRSs in the general PICU population estimate a large effect and therefore upgraded on the basis that confounding is less likely to entirely remove the observed association Unexplained inconsistency (heterogeneous effect sizes) in some populations (e.g., cardiac surgery, transplant) reduces confidence in the causal relationship
	**Limitations:** • Limited predominantly to PICU populations • Limited sample size • Retrospective studies with varied follow up practice • Relevant and appropriate adjustments for confounding variables are yet to be well established • Transportability and heterogeneity of exposure effect not adequately addressed		
Biological gradient	**Indeterminate:** • Studies suggest there is a dose response relationship between AKI severity and chronic kidney sequelae	Benisty (2020), Hessey (2020), Robinson (2021)	Evidence from NRSs is upgraded in GRADE if a dose–response relationship has been observed, as shown in the limited AKI-to-CKD epidemiology
	**Limitations:** • There is a paucity of epidemiological data		
Plausibility	**Biologically plausible:** • Nephron loss, cell cycle arrest, endothelial injury, maladaptive repair mechanisms, inflammation, mitochondrial dysfunction and epigenetic changes all been identified as possible mechanisms of an AKI-to-CKD transition	Ferenbach (2015), He (2017), Sato (2018), Jiang (2020), Sato (2020), Tanemoto (2022)	Evidence downgraded by GRADE as limited adjustment for plausible confounding
Temporality	**Supportive:** • AKI happens months or years before CKD • Biologically plausible mechanism linking AKI and outcome fits within the time course • Pre-exposure prevalence of CKD in the pediatric population is low relative to adult cohorts (less risk of reverse causality)	Hessey (2019), Benisty (2020), Hessey (2020)	Evidence starts as lower quality as no RCTs which by default establish a temporal relationship There are emerging longitudinal pediatric cohort studies which may be able to ensure that the exposure precedes the outcome, allowing upgrading of the evidence
	**Limitations:** • Evidence definitely proving the unidirectional causality of the hypothesis that AKI causes CKD is missing		
Experimentation	**Indeterminate:** • Ongoing generation of evidence drawn from experimental manipulation of AKI and its complications is required		Evidence is downgraded as no effect estimates from randomized studies which are more likely to be causally attributable to the exposure
	**Limitations:** • Pediatric RCTs will be prohibitively expensive, unrepresentative, time-limited, and subject to significant co-intervention over time, making relationships difficult to evaluate • In the absence of RCTs, there is less confidence that the initial conditions are identical in expose and non-exposed		

*AKI* acute kidney injury; *CKD* chronic kidney disease; *PICU* pediatric intensive care; *GRADE* grading of recommendations, assessment, development, and evaluation; *NRS* non randomized study; *RCT* randomized controlled trial

^a^GRADE defines the quality of evidence as the confidence in an estimate of effect (causal relation) from a body of evidence; therefore, the term upgrading is used when this confidence is increased and the term downgrading when this confidence is lowered

### Strength of association and consistency

The stronger an association or correlation is between two variables, the more suggestive it is of a cause/effect relationship. The strength of association between pediatric AKI and short term, in-hospital outcomes strengthens the contention of a causal relationship. Recently, large observational cohort studies have demonstrated that AKI in pediatric critical care populations is strongly associated with poorer hospital outcomes. The Assessment of Worldwide Acute Kidney Injury Epidemiology in Neonates (AWAKEN) [1] study which evaluated more than 2000 neonates with a variety of admission diagnoses, across a wide range of gestational ages, from multiple centers and countries, found that neonates with AKI had four times higher odds of death even after adjusting for severity of illness and other factors; neonates with AKI additionally experienced longer hospital lengths of stay (5 days longer). Another large (*n* = 4 683) multicenter, multinational study in children and young adults, the Assessment of Worldwide Acute Kidney Injury, Renal Angina, and Epidemiology study (AWARE), demonstrated that AKI is independently associated with increased mortality, length of ICU stay, and duration of mechanical ventilation; severe AKI conferred an increased risk of 28-day mortality with an adjusted odds ratio (OR) of 1.77 (95% confidence interval (CI), 1.17–2.68) [2]. The strength of the association is further supported by a recent cohort study of over 1000 children admitted to an ICU in whom severe AKI was associated with greater PICU mortality (adjusted OR, 11.93; 95% CI 4.68–30.42), as well as longer ventilation time and length of stay [18]. Large registry data of children on extracorporeal membrane oxygenation (ECMO) reinforce this causal inference as it demonstrates an association between AKI and mortality of clinically important magnitude which remains statistically significant after adjusting for the multifactorial nature of illness [19]; adjusted OR for mortality following AKI was 3.2 (*p* < 0.0001) and 1.7 (*p* < 0.001) in the neonatal and pediatric cohorts respectively. It is important to note that older and smaller studies have shown similar findings [20–24] and that this viewpoint is supported by adult data. In a recent pooled analysis, using random effects modeling, Girling et al. estimated a 2.3-fold increase in the adjusted risk of death in critically ill adults attributable to AKI, which was statistically significant. Even the lower estimate bound suggested a 30% increased risk of death, which is clinically meaningful given the high prevalence of AKI [25]. In sum, the observed effect of pediatric AKI on short-term survival, hospital stay, and ventilation time is strong and persists after adjusting for potential confounding effects, supporting a causal link between AKI and inferior outcomes as well as the hypothesis that prevention of AKI events could influence outcomes.

Hill’s consistency criterion is upheld when multiple epidemiological studies show a consistent association despite varying populations, epidemiological methods, and locations. Indeed, studies across varied cohorts of children and heterogeneous study populations consistently show that AKI is independently associated with mortality, length of stay, and/or duration of mechanical ventilation. The association is seen in neonates (including low birth weight newborns) and older children, in cardiac and non-cardiac surgical populations, in sepsis and other infectious diseases, in hypoxic ischemic encephalopathy (HIE), ECMO, children with cancer, and following snake envenomation and nephrotoxic medication exposure [1, 2, 19–24, 26–28]. This consistent association is also seen across adult cohorts [25]. The association between pediatric AKI and adverse in-hospital outcomes is also seen when different study designs are used, when controlling for different potentially confounding variables, when examining hospital and community acquired disease, and regardless of whether illness severity scoring includes kidney function criteria [1, 2, 29, 30]. While several consensus definitions of pediatric AKI have been employed over the last two decades, it has been shown, including in a study of almost 150,000 pediatric hospitalizations, that mortality is independently associated with AKI regardless of the definition used [26]. Finally, the independent association between hospital acquired AKI and mortality is seen across different geographical locations around the world and in studies including low-income countries, although this data is not specific to pediatrics [31].

When evaluating the consistency of the relationship between AKI and poorer outcomes, it is important to note that not all AKI is associated with poorer outcomes. Indeed, Sutherland et al. found that while AKI was associated with mortality in ICU settings, this was not true outside of the ICU despite the distribution of AKI severity appearing similar across the two populations [26]. While this might, at first glance, suggest *inconsistency*, we believe this highlights the fact that AKI is a clinical syndrome due to varying underlying pathophysiological processes; it is increasingly recognized that there exist different AKI phenotypes which behave differently, and we should not consider all AKI as equivalent in our causal thinking. Furthermore, these AKI phenotypes exist in various pathophysiologic conditions and the causal impact of AKI is of a continuous, rather than dichotomous nature; while AKI may increase the risk of poor outcomes in all populations, the underlying risk for those outcomes varies across populations and patients.

Although most of our current epidemiological understanding of pediatric AKI’s outcome effects relates to short-term outcomes, pediatric AKI has been implicated for a while as a risk factor for the development of CKD [32–35]. Before this association can be presumed to represent a causal relationship, it is important to assess the strength and consistency of the association. Greenberg et al. systematically reviewed the data available prior to 2014 and found that defining the magnitude and significance of the association between long-term risk of CKD and mortality in children after AKI was problematic; there were ten small cohort studies with a combined total of only 346 patients, variable definitions of AKI were used, there was no comparator group without AKI in any of the published studies, and disparate outcomes were investigated [36]. Similar limitations are seen in a review of older neonatal data evaluating AKI and long-term kidney outcomes [32]. Fortunately, these issues have been less prevalent in more recent studies. A narrative review by Hessey et al. identified eleven studies between 2015 and 2019 examining the association between PICU-AKI and hypertension, proteinuria, and CKD [37]. Four of these studies examined the association of CKD following AKI in general PICU cohorts [38–41]. Patients had mixed illness etiologies and methodologies varied from outcome studies to analyses of administrative datasets. Follow-up time across these studies varied from 2 to 6 years, as did the methods of defining outcomes. However, consistently across all studies, children with PICU-AKI were at higher risk of developing hypertension, proteinuria, and/or CKD; generally, over twice the risk of CKD or hypertension was observed. Six studies in this review reported long-term kidney outcomes following PICU-AKI exclusively in subjects who underwent cardiac surgery [5, 42–46] and one study evaluated children with heart or liver transplant [47]. The strength of the association was variable and not consistent amongst these cohorts. Although the majority of studies looking at the association between pediatric and CKD are based on ICU cohorts, there is some evidence from the non-critical care setting. Menon et al. performed a retrospective cohort study of children with high nephrotoxic medication exposure in a non-critical care setting and found that at 6-month follow-up, the AKI cohort had significantly lower eGFR and a higher prevalence of proteinuria and hypertension; patients with nephrotoxic-associated AKI had almost fourfold higher risk of developing CKD compared with controls [48]. In sum, when applying these fundamental tenets of causal assessment to the extent to which pediatric AKI may be causative in adverse long-term outcomes, there is currently some support but too much divergence and not enough epidemiology to definitively infer causation.

Importantly, observed divergence in the data this far does not necessarily undermine a causal relationship. Similar to observations in short-term outcomes, all AKI does not need to be considered equivalent in our causal thinking; determining the target population to which the results are intended to generalize needs to be a critical decision, for which enough evidence does not yet exist. Larger studies, with more diverse populations, looking at the heterogeneity of exposure effects, effect modification, and the transportability of the causation paradigm between different populations to further elucidate consistency need to be done.

There are additional limitations present in the published evidence that need to be recognized when thinking about strength of association and consistency within this causative framework. Given the nature of AKI and the outcomes being evaluated, factors that may confound the relationship are difficult to eliminate. However, if there is appropriate, reliable, and valid measurement of confounding variables, confounding may be controlled. Confounding variables in the AKI to short-term outcomes relationship are fairly well established and recent large pediatric studies have used appropriate methods to control for such confounders [1, 2, 18, 19]. The viewpoint of strength of association within the AKI to CKD paradigm should also be framed in terms of the effects of confounding; however, relevant and appropriate adjustments for confounding variables are yet to be established. Additional limitations are particularly relevant to the longer term outcome of CKD. Pediatric evidence existing to date still pertains to a limited sample size and given the increasingly recognized heterogeneity in AKI phenotype, this places constraints on the granularity of the associations that can be assessed. In addition, in the absence of definitive knowledge regarding other baseline CKD risk factors, for example, the presence or absence of congenital kidney abnormalities in the majority of studies, larger sample sizes, and population-based studies are critically important. Lastly, given the retrospective nature of many studies and the varied follow-up practice of children with AKI, bias may create challenges in determining true associations.

### Biological gradient

A biological gradient is a relationship between the severity of an exposure and the magnitude of its outcome. Greater exposure to a causative factor or condition should generally lead to greater incidence of the effect. In traditional epidemiology, a monotonic, linear, biological gradient was thought to provide the clearest evidence of a causal relationship. However, it is now acknowledged that this view is an overly simplistic representation of most causal relationships [14, 16]. In the largest multicenter, multinational pediatric cohort study performed to date, a stepwise increase in AKI severity conferred an incremental risk of death, longer ICU stay, and longer duration of ventilation, after adjusting for illness severity [2]. In neonates, similar observations have been made, with higher AKI stages associated with worse outcomes [1]. Although a classic dose response relationship with mortality was not observed in the AWAKEN study at the lower stages of AKI, severe AKI had higher mortality than less severe, and length of stay did have an incremental dose–response relationship with AKI severity [1]. Support for the viewpoint of a biological gradient can also be drawn from studies that define “dose” outside the confines of the currently accepted three-stage AKI severity classification. When pediatric AKI severity is scored from 1 to 6 by combining the serum creatinine and urine output-based stages, a progressive increase in mortality and ICU length of stay is seen as this composite AKI score increases [49]. AKI of longer duration (i.e., persistent vs. transient) is associated with increased risk of mortality, longer duration of ventilation, and longer hospital stay [18]. Additionally, intra-patient progression of AKI severity is associated with higher risk of poor outcomes [50]. Repeat episodes of AKI during the same admission are associated with worse outcomes in children, including increased mortality, longer length of hospital stay, and longer ventilation time [51]. Adult epidemiology also supports the viewpoint that adverse outcomes increase with increasing AKI severity [25, 52]; in the largest adult study of over 300,000 patients, a clear stepwise gradient across AKI stages was observed which was statistically significant between all groups [30]. Overall, data support the presence of a biological gradient between the severity of AKI and the magnitude of short-term negative outcomes.

It is tempting to look for a biological gradient in the association between pediatric AKI and longer term kidney outcomes; however, the paucity of epidemiological data makes this challenging. That said, early evidence does suggest that such a gradient exists. Benisty et al. [39] evaluated a prospective cohort of children 6 years after PICU admission and found that any AKI and severe AKI (stage 2 or 3) were associated with a 2.2 (95% CI 1.1–4.4) and 6.6 (95% CI 1.5–28.3) higher adjusted OR for CKD and pre-hypertension or worse, respectively (*n* = 277). This incremental increased risk of negative long-term kidney outcome with increased severity of AKI was also observed in a larger study (*n* = 1978) of children using multicenter administrative data [40]. Given that in many studies children with dialysis-treated AKI (i.e., most severe) are under-represented, Robinson et al. performed a large population-based retrospective-matched cohort study of dialysis-treated AKI survivors [3]. They found that pediatric dialysis-treated AKI survivors were at significantly increased long-term risks of kidney failure, death, CKD, and hypertension, versus hospitalized comparators. At > 5 years post-discharge, dialysis-treated AKI survivors were at increased risk of MAKE (major adverse kidney events), defined as a composite of all-cause mortality, kidney failure, or de novo CKD (adjusted HR 1.89; 95% CI 1.31–2.73) [3]. Many adult studies have also described the association between AKI and progression to CKD and meta-analysis of such studies has demonstrated that not only is there a strong association between AKI and the development of CKD, but the risk of CKD increases in a graded fashion with AKI severity [53]. In adults, trying to understand the relationship between AKI and CKD has been quite complex, with an argument made that the magnitude of the risk and gradient across stages likely depends on the residual kidney function, premorbid conditions, and repair capacity after kidney stress [54]. However, in a large diabetic cohort, in-hospital AKI increased the risk for stage 4 CKD (hazard ratio (HR), 3.56; 95% CI 2.76–4.61), and importantly, when considering causation criteria, each episode of AKI doubled the risk. These observations occurred independent of other major risk factors of progression, including baseline kidney function, comorbidities, and proteinuria. Thus, while there is a paucity of data exploring the biologic gradient between AKI and long-term kidney outcomes in children, the studies currently available and those in adults do suggest that there is a dose response relationship between AKI severity and chronic kidney sequelae.

### Plausibility

A biologically plausible mechanism of action linking exposure and effect strengthens the argument for causality. Although plausibility is a component of the framework for causal inference in epidemiologic studies, this viewpoint is complex as it implies data integration of non-epidemiological studies and necessitates the incorporation of our current biologic understanding. There is an evolving recognition that there is variation in the nature of AKI at the genetic, molecular, and cellular level, implying that more than one biological model underpins the link between AKI and its associated outcomes. Furthermore, although causal inference historically was based upon the assumption of single-factor direct relationships, it is now understood that the interplay between multiple factors determines disease outcomes. As such, demonstrating the biological plausibility of a causal relationship can be complex.

A simplistic view of the pediatric AKI exposure to adverse in-hospital outcome paradigm is that the established AKI sequelae including uremic toxin accumulation, metabolic acidosis, hyperkalemia, and fluid overload are accepted mechanisms of mortality and morbidity. Epidemiological data support this rationale, with a large proportion of the excess risk of negative short-term outcomes attributable in statistical modeling to metabolic and fluid related complications [55]. However, correction of these sequelae via supportive therapy does not eliminate risk [55], likely because such therapies do not correct the underlying pathophysiology. Over the last decade, the concept of organ cross-talk during kidney injury has evolved and cellular adhesion molecules, lymphocyte trafficking, generation of oxidative stress, endothelial injury, and release of pro-inflammatory cytokines and chemokines have been cited as mechanisms of remote organ injury and modification of immune response systems [56–59]. Clinical and experimental findings have illuminated a complex but plausible exposure-to-effect paradigm for the causal relationship between AKI and the negative short-term outcomes observed.

There is increasing understanding of the biological plausibility for longer term kidney outcomes as well. Nephron loss, cell cycle arrest, endothelial injury, maladaptive repair mechanisms, inflammation, mitochondrial dysfunction, and epigenetic changes have all been identified as possible mechanisms of an AKI to CKD transition [60–64]. A mechanistic gap that has existed in the AKI to CKD paradigm is the explanation of how a transition can occur from AKI to CKD after functional recovery from injury has occurred. This gap is being addressed by the field of epigenetics; epigenetic memory is the ability of cells to retain and transmit changes in gene expression patterns induced by preceding environmental stimuli to their daughter cells [65, 66]. It is well recognized now that acutely acquired epigenetic alterations can predispose one to late-onset diseases [67]. It is biologically plausible that the “memory” of AKI leads to CKD. In the last decade, there has been a tremendous amount of basic science work demonstrating epigenetic alterations induced by AKI; although, there is currently insufficient data available on the retained epigenetic modifications (“epigenetic memory”) of AKI that may contribute to the progression to CKD [68]. Hill’s plausibility criterion requires that the association between AKI and CKD be explained in the presence of existing biological models, with causation being a continuum from highly unlikely to highly likely. Given the current evidence, it is likely that epigenetic alterations that cause pro-regenerative phenotypic changes during injury repair maladaptively remain in response to severe or repetitive insults, leading to progressive tubulointerstitial fibrosis.

The viewpoint of biological plausibility strongly supports the exposure-to-effect paradigm for AKI and CKD; however, there are concepts identified in adult populations which introduce some uncertainty. Some potential problems with regard to plausibility include the shared risk factors between AKI and CKD, the possibility of misclassification of AKI as a discrete event rather than progression of pre-existing kidney disease, and misclassification of outcome (de novo CKD versus CKD progression). Importantly, pediatric research and clinical epidemiology may be the key to addressing these concerns as the ideal biologic plausibility model for causality requires that healthy individuals sustain AKI to assess whether there is de novo chronically sustained disease of the kidney.

### Temporality

For an exposure-outcome relationship to be causal, there must be a temporal progression between the two measures; the alleged effect must follow the suspected cause and not the other way around. In addition, the biologically plausible mechanism linking the two must fit within this time course. When making a causal assessment of the in-hospital outcomes following pediatric AKI, the temporal sequence supports causation. AKI comes before death, extubation, and discharge; the AKI exposure occurs early in the course. In the AWAKEN study, AKI occurred most commonly in the first week of life and the majority of mortality occurred in the first 50 days of hospital admission [1]. In the general ICU pediatric population, persistent AKI is most likely to have its onset within 24 h of PICU admission and is associated with longer ventilation and hospital length of stay [69]. The biological mechanisms discussed above fit appropriately within time frames reported for outcomes in the epidemiological literature.

In pediatrics, temporality is perhaps the easiest of the viewpoints to satisfy in evaluating the relationship between AKI and the long-term outcome of CKD. Most if not all studies in children exclude subjects with baseline kidney disease and the prevalence of CKD in the pediatric population is low relative to adult cohorts. In the current pediatric studies, which demonstrate an association between AKI exposure and long-term kidney outcomes, the temporal progression from exposure to outcome is persuasive in causal inference [1, 2, 19–24, 26–28].

### Experimentation

Evidence drawn from experimental manipulation may lead to strong support for causal inference. It is important to note however that in causal assessment, a hierarchical or weighted interpretation of the different viewpoints was not the intended purpose [14]. Evidence from randomized controlled trials (RCTs) is often considered to provide a higher level of certainty in establishing causation than evidence from other epidemiological studies, although the accuracy of this has been critiqued [70]. No RCTs in children have evaluated the manipulation of AKI through a viable preventive strategy and its association with patient outcome, thus causal association cannot be supported in this way. However, this raises the question of whether an RCT in pediatric AKI is necessary to allow for causal inference. It is simplistic to conclude that an RCT designed to show a causative relationship between higher AKI and mortality, for example, by manipulating the AKI exposure with an intervention will result in absolute certainty. It is very likely, however, that we will still not be able to discern with certainty if the changes in outcome are due to the prevention of AKI or to some other effect of the intervention. There need not be an a priori conviction that observational studies and the other causal thinking viewpoints are inferior to RCTs; each method should be considered as capable of providing relevant information. For long-term outcomes, it is even less likely that RCTs will be able to help demonstrate a causal association for AKI. Pediatric RCTs for long-term outcomes, with experimental manipulation of AKI events, will be prohibitively expensive, unrepresentative, time-limited, and subject to significant co-intervention over time, making relationships difficult to evaluate. In the absence of randomization however, confidence that the initial conditions before or at the time of AKI are identical in the AKI-exposed and non-exposed group is challenged; there may be risk factors that are common to both AKI predisposition and CKD predisposition that explain the association, which is particularly troublesome in adult studies [71]. Most AKI studies in children however involve a previously healthy population, where risk factors for CKD present before AKI are far less common than in adults.

Although no RCTs have yet evaluated the outcome effects of experimental manipulation of AKI occurrence in children, there is some evidence that mitigating established AKI sequelae may change outcomes, providing surrogate information that preventing AKI could modify outcomes. Earlier intervention in children to manage the fluid complications of AKI has been observed in some studies to improve in-hospital outcomes. Goldstein et al. demonstrated that implementation of a clinical decision support tool aimed at risk stratifying young ICU patients at high risk of AKI and guiding management of AKI complications (fluid overload) may result in shorter length of ICU [72]. Kwiatkowski et al. demonstrated that elective placement of a peritoneal dialysis catheter for fluid balance management at the time of surgery in infants undergoing cardiac surgery determined to be high risk for AKI, resulted in earlier time to negative fluid balance and was associated with earlier extubation, improved inotrope scores, and fewer electrolyte imbalances [73]. A small (*n* = 22) RCT of neonates undergoing Norwood procedure did not replicate this improvement in outcomes; however, time to negative fluid balance was the same between intervention and control group [74]. A very recent systematic review of both observational and RCT data looking at early peritoneal dialysis catheter placement in children undergoing cardiac surgery and effect on outcomes demonstrated that there is currently significant heterogeneity in the evidence [75]. Pooled analysis showed no association between prophylactic PD catheter placement and in-hospital mortality, and there were mixed results for ICU length of stay, with some studies showing shortened duration associated with use of prophylactic PD catheter insertion and others showing no difference [75]. Assessment of fluid complications also showed variable results, which, when combined with the high risk of bias for many of the studies, highlights that the interventional or experimental data in pediatrics regarding the effect of mitigating established AKI sequelae on outcomes is currently inconsistent.

Adult data is starting to lend some support to causal inference from the viewpoint of experimentation. Early biomarker-based prediction of imminent AKI followed by implementation of the KDIGO care bundle (early optimization of fluid status, maintenance of perfusion pressure, discontinuation of nephrotoxic agents) in cardiac surgery patients was shown to reduce the incidence and severity of AKI, however was not adequately powered to demonstrate changes in short-term outcomes [76]. Subsequently, in a large multicenter RCT, similar risk-based implementation of the same care bundle reduced AKI severity and postoperative creatinine increase and also decreased length of ICU and hospital stay after major noncardiac surgery [77]. Ongoing generation of evidence drawn from experimental manipulation of AKI and its complications in both adults and children may further strengthen support within causality assessment.

### Specificity, analogy, and coherence

The evaluation of causality thus far has focused on strength of association, consistency, biological gradient, plausibility, temporality, and experimentation, as evidence suggests that the viewpoints of specificity, analogy, and coherence may have limited usefulness in modern causal assessment [15]. According to the Bradford Hill viewpoints, a relationship is specific if the exposure is associated with the outcome in question and no others, and if the outcome is associated with the exposure in question and no others. It is emphasized in the application of causality frameworks that a non-specific relationship does not undermine causality. This is because specificity is rare in epidemiology; in human health and disease, including AKI, multiple causations (where one exposure may affect many outcomes and one outcome may be affected by many exposures) limit the utility of directly applying specificity in epidemiological practice. The application of the analogy consideration is equally limited in its utility [15, 78]. Hill implied that in some circumstances it is fair to judge causality by analogy whereby prior knowledge and patterns can be used to infer similar causal associations. In this time of vast and accessible knowledge, even with just slight creativity, analogy could be identified for every situation and the importance has likely been diluted since the inception of these criteria. Although it may engender a sense of credibility through the resemblance of other accepted truths, analogy is not necessary in arguing causation [16], and we have therefore declined to include it in this assessment of causal strength. Finally, coherence is the assessment of how a putative relationship fits into existing theory and empirical evidence and is poorly delineated from plausibility [15]. A causal conclusion should not fundamentally contradict present substantive knowledge. In current day epidemiology, studies examining AKI and outcomes essentially emerge from existing theory; hence, it is not a viewpoint that is helpful in establishing causality.

## Conclusions

Available data in children support a causal link between AKI and a limited scope of short-term outcomes including mortality, length of stay, and ventilation time. This is based upon the viewpoints of strength of association, consistency of findings, temporality, presence of a biologic gradient, and plausibility, and supports the hypothesis that prevention and mitigation of AKI is likely to improve early outcomes in children.

Insufficient data currently exists to demonstrably state that a causal relationship exists between AKI and longer term health outcomes. Clarifying the causal nature of longer term associations requires further high-quality observational studies in children (with relevant comparator cohorts and much larger study samples), careful consideration of what defines the most meaningful and measurable longer term outcomes after pediatric AKI, and integration of evolving biological data related to mechanisms of disease. In evaluating the AKI to CKD paradigm within a causal framework, it will be important to understand which children are at risk of both AKI and CKD. In addition, to better establish temporal progression, the metrics for ongoing injury and repair need to evolve as does the identification of optimal follow-up time.

Epidemiological evidence surrounding the causative link between AKI and CKD in the population is imperfect and complex. Two things are of paramount importance to move this field forward. First is to focus on pediatric AKI. The previous belief that AKI in children has negligible health impact [71] and has been discarded due to the evidence which has accumulated over the last decade. More importantly, children provide a unique opportunity to evaluate the AKI to CKD transition because they do not have the underlying comorbidities that make adult epidemiology so complex. Second, biological plausibility is an increasingly important criterion in evidence-based decision-making related to AKI as evidence will likely continue to come from observational rather than controlled studies. There needs to be further elucidation of molecular and cellular mechanisms involved in maladaptive recovery and repair processes underpinning the AKI to CKD paradigm.

It is important to note that when the Bradford Hill criteria first emerged, mechanistic connections between exposure and disease were not well understood and traditional epidemiological studies treated the connection between exposure and disease/outcome as a “black box.” We now have new and more diverse types of information beyond the traditional epidemiologic study designs that were available when Hill Criteria were first written. It is therefore important not to take a singular approach but to incorporate data from a multitude of sources to answer this question. Researchers can now evaluate the causal relationship by illuminating various mechanistic points along the exposure-to-effect paradigm, creating a consistent story across multiple disciplines including in vitro studies, animal models, and molecular experimentations [16]. In addition, although we have used the Bradford Hill viewpoints to frame our causal thinking here, it is important to consider that over time, new approaches are evolving, such as complex systems dynamic methods, which can integrate our growing knowledge and integrate a life course perspective and may offer a solutions to some of the challenges AKI epidemiology faces in the realm of causal thinking [79].

**Fig. 2:**
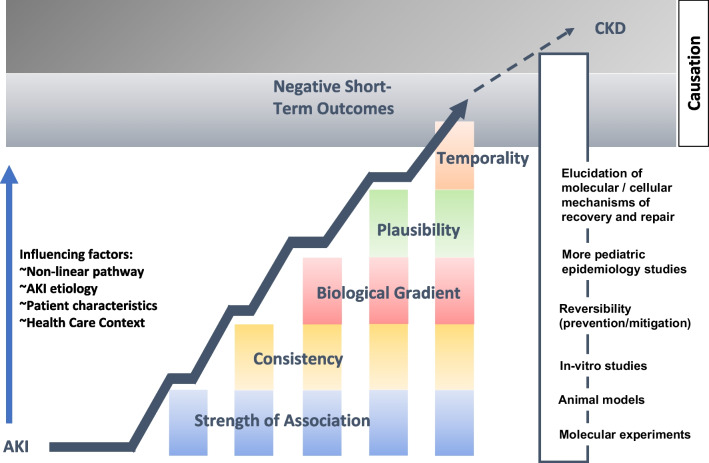
Cumulative contribution of foundational viewpoints and novel information to causal inference in the AKI to negative health outcome paradigm. The causative pathway from AKI to negative outcomes is non-linear with AKI etiology, patient characteristics, and healthcare context affecting the causative trajectory. A causal link between AKI and a limited scope of short-term outcomes including mortality, length of stay, and ventilation time is supported by the viewpoints of strength of association, consistency of findings, temporality, presence of a biologic gradient, and plausibility. There is a need to incorporate the concept of reversibility as well as mechanistic points along the exposure-to-effect paradigm, and create a consistent story using in vitro studies, animal models, and molecular experimentation to be able to extend causative thinking further towards long-term outcomes. AKI, acute kidney disease; CKD, chronic kidney disease

In summary, when evaluating the current pediatric AKI epidemiological data in the context of causal inference, the picture is far from complete (Fig. 2). The causative pathway from AKI to negative outcomes is likely non-linear, and different aspects including etiology, patient characteristics, and healthcare context, will affect causative trajectories during childhood. We may find that it is important to incorporate the concept of *Reversibility* into more traditional causal analysis frameworks. Preventing or mitigating AKI, assuming a causal relationship with negative outcomes, should lead to improved outcomes. Demonstrating this will solidify confidence in the causal relationship, improve child health, and highlight an aspect which is highly relevant to clinicians, scientists, and policy makers. Perhaps most importantly, questions need to be asked regarding what the most meaningful outcomes are and to whom, as well as how to best measure these outcomes. Else, proving causality is of little importance.

## Supplementary Information

Below is the link to the electronic supplementary material.

Graphical abstract (PPTX 117 KB)

## References

[1] Jetton JG, Boohaker LJ, Sethi SK, Wazir S, Rohatgi S, Soranno DE et al (2017) Incidence and outcomes of neonatal acute kidney injury (AWAKEN): a multicentre, multinational, observational cohort study. Lancet Child Adolesc Health 1:184–194. 10.1016/S2352-4642(17)30069-X 29732396 10.1016/S2352-4642(17)30069-X PMC5933049

[2] Kaddourah A, Basu RK, Bagshaw SM, Goldstein SL (2017) Epidemiology of acute kidney injury in critically ill children and young adults. N Engl J Med 376:11–20. 10.1056/NEJMoa1611391 27959707 10.1056/NEJMoa1611391 PMC5322803

[3] Robinson CH, Jeyakumar N, Luo B et al (2021) Long-term kidney outcomes following dialysis-treat childhood acute kidney injury: a population-based cohort study. J Am Soc Nephrol 32:2005–2019. 10.1681/ASN.2020111665 34039667 10.1681/ASN.2020111665 PMC8455253

[4] Greenberg JH, Zappitelli M, Devarajan P, TRIBE-AKI Consortium et al (2016) Kidney outcomes 5 years after pediatric cardiac surgery: the TRIBE-AKI study. JAMA Pediatr 170:1071–1078. 10.1001/jamapediatrics.2016.1532 27618162 10.1001/jamapediatrics.2016.1532 PMC5476457

[5] Madsen NL, Goldstein SL, Frøslev T, Christiansen CF, Olsen M (2017) Cardiac surgery in patients with congenital heart disease is associated with acute kidney injury and the risk of chronic kidney disease. Kidney Int 92:751–756. 10.1016/j.kint.2017.02.021 28412020 10.1016/j.kint.2017.02.021

[6] Akcan-Arikan A, Gebhard DJ, Arnold MA, Loftis LL, Kennedy CE (2017) Fluid overload and kidney injury score: a multidimensional real-time assessment of renal disease burden in the critically ill patient. Pediatr Crit Care Med 18:524–530. 10.1097/PCC.0000000000001123 28406863 10.1097/PCC.0000000000001123

[7] Abbasi A, Mehdipour Rabori P, Farajollahi R, Mohammed Ali K, Ataei N, Yousefifard M, Hosseini M (2020) Discriminatory precision of renal angina index in predicting acute kidney injury in children; a systematic review and meta-analysis. Arch Acad Emerg Med 8:e39 32259128 PMC7130445

[8] Gist KM, Goldstein SL, Wrona J, Alten JA, Basu RK, Cooper DS, Soranno DE, Duplantis J, Altmann C, Gao Z, Faubel S (2017) Kinetics of the cell cycle arrest biomarkers (TIMP-2*IGFBP-7) for prediction of acute kidney injury in infants after cardiac surgery. Pediatr Nephrol 32:1611–1619. 10.1007/s00467-017-3655-y 28382566 10.1007/s00467-017-3655-y

[9] Menon S, Goldstein SL, Mottes T, Fei L, Kaddourah A, Terrell T, Arnold P, Bennett MR, Basu RK (2016) Urinary biomarker incorporation into the renal angina index early in intensive care unit admission optimizes acute kidney injury prediction in critically ill children: a prospective cohort study. Nephrol Dial Transplant 31:586–594. 10.1093/ndt/gfv457 26908772 10.1093/ndt/gfv457 PMC6281075

[10] Sutherland SM, Alobaidi R, Gorga SM et al (2024) Epidemiology of acute kidney injury in children: a report from the 26th Acute Disease Quality Initiative (ADQI) consensus conference. Pediatr Nephrol 39:919–928. 10.1007/s00467-023-06164-w 37874357 10.1007/s00467-023-06164-w PMC10817829

[11] Goldstein SL, Akcan-Arikan A, Alobaidi R et al (2022) Consensus-based recommendations on priority activities to address acute kidney injury in children: a modified Delphi consensus statement. JAMA Netw Open 5:e2229442. 10.1001/jamanetworkopen.2022.29442 36178697 10.1001/jamanetworkopen.2022.29442 PMC9756303

[12] Hodgson LE, Selby N, Huang TM, Forni LG (2019) The role of risk prediction models in prevention and management of AKI. Semin Nephrol 39:421–430. 10.1016/j.semnephrol.2019.06.002 31514906 10.1016/j.semnephrol.2019.06.002

[13] Meersch M, Schmidt C, Hoffmeier A et al (2017) Prevention of cardiac surgery-associated AKI by implementing the KDIGO guidelines in high risk patients identified by biomarkers:the PrevAKI randomized controlled trial. Intensive Care Med 43:1551–1561. 10.1007/s00134-016-4670-3 28110412 10.1007/s00134-016-4670-3 PMC5633630

[14] Hill AB (2015) The environment and disease: association or causation? JR Soc Med 108:32–37. 10.1177/0141076814562718 10.1177/0141076814562718 PMC4291332 25572993

[15] Shimonovich M, Pearce A, Thomson H, Keyes K, Katikireddi SV (2021) Assessing causality in epidemiology: revisiting Bradford Hill to incorporate developments in causal thinking. Eur J Epidemiol 36:873–887. 10.1007/s10654-020-00703-7 33324996 10.1007/s10654-020-00703-7 PMC8206235

[16] Fedak KM, Bernal A, Capshaw ZA, Gross S (2015) Applying the Bradford Hill criteria in the 21st century: how data integration has changed causal inference in molecular epidemiology. Emerg Themes Epidemiol 12:14. 10.1186/s12982-015-0037-4 26425136 10.1186/s12982-015-0037-4 PMC4589117

[17] Schünemann H, Hill S, Guyatt G et al (2011) The GRADE approach and Bradford Hill’s criteria for causation. J Epidemiol Community Health 65:392–395. 10.1136/jech.2010.119933 20947872 10.1136/jech.2010.119933

[18] Alobaidi R, Morgan C, Goldstein SL, Bagshaw SM (2020) Population-based epidemiology and outcomes of acute kidney injury in critically ill children. Pediatr Crit Care Med 21:82–91. 10.1097/PCC.0000000000002128 31568261 10.1097/PCC.0000000000002128

[19] Askenazi DJ, Ambalavanan N, Hamilton K, Cutter G, Laney D, Kaslow R et al (2011) Acute kidney injury and renal replacement therapy independently predict mortality in neonatal and pediatric noncardiac patients on extracorporeal membrane oxygenation. Pediatr Crit Care Med 12:e1-6. 10.1097/PCC.0b013e3181d8e348 20351617 10.1097/PCC.0b013e3181d8e348

[20] Li S, Krawczeski CD, Zappitelli M, Devarajan P, Thiessen-Philbrook H, Coca SG et al (2011) Incidence, risk factors, and outcomes of acute kidney injury after pediatric cardiac surgery: a prospective multicenter study. Crit Care Med 39:1493–1499. 10.1097/CCM.0b013e31821201d3 21336114 10.1097/CCM.0b013e31821201d3 PMC3286600

[21] Blinder JJ, Goldstein SL, Lee VV, Baycroft A, Fraser CD, Nelson D et al (2012) Congenital heart surgery in infants: effects of acute kidney injury on outcomes. J Thorac Cardiovasc Surg 143:368–374. 10.1016/j.jtcvs.2011.06.021 21798562 10.1016/j.jtcvs.2011.06.021

[22] Selewski DT, Jordan BK, Askenazi DJ, Dechert RE, Sarkar S (2013) Acute kidney injury in asphyxiated newborns treated with therapeutic hypothermia. J Pediatr 162:725-729.e1. 10.1016/j.jpeds.2012.10.002 23149172 10.1016/j.jpeds.2012.10.002

[23] Alkandari O, Eddington KA, Hyder A, Gauvin F, Ducruet T, Gottesman R et al (2011) Acute kidney injury is an independent risk factor for pediatric intensive care unit mortality, longer length of stay and prolonged mechanical ventilation in critically ill children: a two-center retrospective cohort study. Crit Care 15:R146. 10.1186/cc10269 21663616 10.1186/cc10269 PMC3219018

[24] Schneider J, Khemani R, Grushkin C, Bart R (2010) Serum creatinine as stratified in the RIFLE score for acute kidney injury is associated with mortality and length of stay for children in the pediatric intensive care unit. Crit Care Med 38:933–939. 10.1097/CCM.0b013e3181cd12e1 20124891 10.1097/CCM.0b013e3181cd12e1

[25] Girling BJ, Channon SW, Haines RW, Prowle JR (2020) Acute kidney injury and adverse outcomes of critical illness: correlation or causation? Clin Kidney J 13:133–141. 10.1093/ckj/sfz158 32296515 10.1093/ckj/sfz158 PMC7147312

[26] Sutherland SM, Byrnes JJ, Kothari M, Longhurst CA, Dutta S, Garcia P et al (2015) AKI in hospitalized children: comparing the pRIFLE, AKIN, and KDIGO definitions. Clin J Am Soc Nephrol 10:554–561. 10.2215/CJN.01900214 25649155 10.2215/CJN.01900214 PMC4386245

[27] Searns JB, Gist KM, Brinton JT, Pickett K, Todd J, Birkholz M et al (2020) Impact of acute kidney injury and nephrotoxic exposure on hospital length of stay. Pediatr Nephrol 35:799–806. 10.1007/s00467-019-04431-3 31940070 10.1007/s00467-019-04431-3

[28] Sarkar S, Sinha R, Chaudhury AR, Maduwage K, Abeyagunawardena A, Bose N et al (2021) Snake bite associated with acute kidney injury. Pediatr Nephrol 36:3829–3840. 10.1007/s00467-020-04911-x 33559706 10.1007/s00467-020-04911-x

[29] Barrantes F, Tian J, Vazquez R, Amoateng-Adjepong Y, Manthous CA (2008) Acute kidney injury criteria predict outcomes of critically ill patients. Crit Care Med 36:1397–1403. 10.1097/CCM.0b013e318168fbe0 18434915 10.1097/CCM.0b013e318168fbe0

[30] Hsu CN, Chen HL, Tain YL (2018) Epidemiology and outcomes of community-acquired and hospital-acquired acute kidney injury in children and adolescents. Pediatr Res 83:622–629. 10.1038/pr.2017.262 29155805 10.1038/pr.2017.262

[31] Mehta RL, Burdmann EA, Cerdá J, Feehally J, Finkelstein F, García-García G et al (2016) Recognition and management of acute kidney injury in the International Society of Nephrology 0by25 Global Snapshot: a multinational cross-sectional study. Lancet 387:2017–2025. 10.1016/S0140-6736(16)30240-9 27086173 10.1016/S0140-6736(16)30240-9

[32] Chaturvedi S, Ng KH, Mammen C (2017) The path to chronic kidney disease following acute kidney injury: a neonatal perspective. Pediatr Nephrol 32:227–241. 10.1007/s00467-015-3298-9 26809804 10.1007/s00467-015-3298-9

[33] Sigurjonsdottir VK, Chaturvedi S, Mammen C, Sutherland SM (2018) Pediatric acute kidney injury and the subsequent risk for chronic kidney disease: is there cause for alarm? Pediatr Nephrol 33:2047–2055. 10.1007/s00467-017-3870-6 29374316 10.1007/s00467-017-3870-6

[34] Hoy WE, White AV, Dowling A, Sharma SK, Bloomfield H, Tipiloura BT, Swanson CE, Mathews JD, McCredie DA (2012) Post-streptococcal glomerulonephritis is a strong risk factor for chronic kidney disease in later life. Kidney Int 81:1026–1032. 10.1038/ki.2011.478 22297679 10.1038/ki.2011.478

[35] Garg AX, Suri RS, Barrowman N, Rehman F, Matsell D, RosasArellano MP, Salvadori M, Haynes RB, Clark WF (2003) Longterm renal prognosis of diarrhea-associated hemolytic uremic syndrome: a systematic review, meta-analysis, and meta-regression. JAMA 290:1360–1370. 10.1001/jama.290.10.1360 12966129 10.1001/jama.290.10.1360

[36] Greenberg JH, Coca S, Parikh CR (2014) Long-term risk of chronic kidney disease and mortality in children after acute kidney injury: a systematic review. BMC Nephrol 15:184. 10.1186/1471-2369-15-184 25416588 10.1186/1471-2369-15-184 PMC4251927

[37] Hessey E, Melhem N, Alobaidi R, Ulrich E, Morgan C, Bagshaw SM, Sinha MD (2021) Acute kidney injury in critically ill children is not all acute: lessons over the last 5 years. Front Pediatr 9:648587. 10.3389/fped.2021.648587 33791260 10.3389/fped.2021.648587 PMC8005629

[38] Hessey E, Perreault S, Dorais M, Roy L, Zappitelli M (2019) Acute kidney injury in critically ill children and subsequent chronic kidney disease. Can J Kidney Health Dis 6:2054358119880188. 10.1177/2054358119880188 31662875 10.1177/2054358119880188 PMC6794652

[39] Benisty K, Morgan C, Hessey E, Huynh L, Joffe AR, Garros D et al (2020) Kidney and blood pressure abnormalities 6 years after acute kidney injury in critically ill children: a prospective cohort study. Pediatr Res 88:271–278. 10.1038/s41390-019-0737-5 31896128 10.1038/s41390-019-0737-5

[40] Hessey E, Perreault S, Roy L, Dorais M, Samuel S, Phan V et al (2020) Acute kidney injury in critically ill children and 5-year hypertension. Pediatr Nephrol 35:1097–1107. 10.1007/s00467-020-04488-5 32162099 10.1007/s00467-020-04488-5

[41] Al-Otaibi NG, Zeinelabdin M, Shalaby MA, Khathlan N, Mashat GD, Zahrani AA, NoorSaeed SM, Shalabi NM, Alhasan KA, Sharief SN, Albanna AS, Kari JA (2017) Impact of acute kidney injury on long-term mortality and progression to chronic kidney disease among critically ill children. Saudi Med J 38:138–142. 10.15537/smj.2017.2.16012 28133685 10.15537/smj.2017.2.16012 PMC5329624

[42] Cooper DS, Claes D, Goldstein SL, Bennett MR, Ma Q, Devarajan P, Krawczeski CD (2016) Follow-up renal assessment of injury long-term after acute kidney injury (FRAIL-AKI). Clin J Am Soc Nephrol 11:21–29. 10.2215/CJN.04240415 26576618 10.2215/CJN.04240415 PMC4702230

[43] Greenberg JH, Devarajan P, Thiessen-Philbrook HR, Krawczeski C, Parikh CR, Zappitelli M, TRIBE-AKI Consortium (2018) Kidney injury biomarkers 5 years after AKI due to pediatric cardiac surgery. Pediatr Nephrol 33:1069–1077. 10.1007/s00467-018-3888-4 29511889 10.1007/s00467-018-3888-4 PMC5945328

[44] Huynh L, Rodriguez-Lopez S, Benisty K, Dancea A, Garros D, Hessey E, Joffe A, Joffe R, Mackie A, Palijan A, Paun A, Pizzi M, Zappitelli M, Morgan C (2020) Follow-up after neonatal heart disease repair: watch out for chronic kidney disease and hypertension! Pediatr Nephrol 35:2137–2145. 10.1007/s00467-020-04621-4 32500246 10.1007/s00467-020-04621-4 PMC7515960

[45] Zappitelli M, Parikh CR, Kaufman JS, Go AS, Kimmel PL, Hsu CY, Coca SG, Chinchilli VM, Greenberg JH, Moxey-Mims MM, Ikizler TA, Cockovski V, Dyer AM, Devarajan P, ASsessment, Serial Evaluation, and Subsequent Sequelae in Acute Kidney Injury (ASSESS-AKI) Investigators (2020) Acute kidney injury and risk of CKD and hypertension after pediatric cardiac surgery. Clin J Am Soc Nephrol 15:1403–1412. 10.2215/CJN.00150120 32948644 10.2215/CJN.00150120 PMC7536759

[46] Hollander SA, Montez-Rath ME, Axelrod DM, Krawczeski CD, May LJ, Maeda K, Rosenthal DN, Sutherland SM (2016) Recovery from acute kidney injury and CKD following heart transplantation in children, adolescents, and young adults: a retrospective cohort study. Am J Kidney Dis 68:212–218. 10.1053/j.ajkd.2016.01.024 26970941 10.1053/j.ajkd.2016.01.024

[47] Menon S, Pollack AH, Sullivan E, Murphy T, Smith J (2020) Acute kidney injury and chronic kidney disease after non-kidney solid organ transplantation. Pediatr Transplant 24:e13753. 10.1111/petr.13753 32497381 10.1111/petr.13753

[48] Menon S, Kirkendall ES, Nguyen H, Goldstein SL (2014) Acute kidney injury associated with high nephrotoxic medication exposure leads to chronic kidney disease after 6 months. J Pediatr 165:522-527.e2. 10.1016/j.jpeds.2014.04.058 24928698 10.1016/j.jpeds.2014.04.058

[49] Sutherland SM, Kaddourah A, Gillespie SE, Soranno DE, Woroniecki RP, Basu RK et al (2021) Cumulative application of creatinine and urine output staging optimizes the kidney disease: improving global outcomes definition and identifies increased mortality risk in hospitalized patients with acute kidney injury. Crit Care Med 49:1912–1922. 10.1097/CCM.0000000000005073 33938717 10.1097/CCM.0000000000005073

[50] Sanchez-Pinto LN, Goldstein SL, Schneider JB, Khemani RG (2015) Association between progression and improvement of acute kidney injury and mortality in critically ill children. Pediatr Crit Care Med 16:703–710. 10.1097/PCC.0000000000000461 26132741 10.1097/PCC.0000000000000461

[51] Moffett BS, Arikan AA (2022) Trajectory of AKI in hospitalized pediatric patients—impact of duration and repeat events. Nephrol Dial Transplant 37:1443–1450. 10.1093/ndt/gfab219 34245299 10.1093/ndt/gfab219

[52] Thakar CV, Christianson A, Freyberg R, Almenoff P, Render ML (2009) Incidence and outcomes of acute kidney injury in intensive care units: a Veterans Administration study. Crit Care Med 37:2552–2558. 10.1097/CCM.0b013e3181a5906f 19602973 10.1097/CCM.0b013e3181a5906f

[53] Coca SG, Singanamala S, Parikh C (2012) Chronic kidney disease after acute kidney injury: a systematic review and meta-analysis. Kidney Int 81:442–448. 10.1038/ki.2011.379 22113526 10.1038/ki.2011.379 PMC3788581

[54] Fortrie G, de Geus HRH, Betjes MGH (2019) The aftermath of acute kidney injury: a narrative review of long-term mortality and renal function. Crit Care 23:24. 10.1186/s13054-019-2314-z 30678696 10.1186/s13054-019-2314-z PMC6346585

[55] Libório AB, Leite TT, Neves FMdeO, Teles F, Bezerra CTdeM (2015) AKI complications in critically ill patients: association with mortality rates and RRT. Clin J Am Soc Nephrol 10:21–28. 10.2215/CJN.04750514 25376761 10.2215/CJN.04750514 PMC4284413

[56] White LE, Hassoun HT (2012) Inflammatory mechanisms of organ crosstalk during ischemic acute kidney injury. Int J Nephrol 2012:505197. 10.4061/2012/505197 21826270 10.4061/2012/505197 PMC3118535

[57] Leaf DE (2019) Introduction: Cross-talk between the kidneys and remote organ systems in AKI. Semin Nephrol 39:1–2. 10.1016/j.semnephrol.2018.10.010 30606402 10.1016/j.semnephrol.2018.10.010

[58] Pickkers P, Darmon M, Hoste E, Joannidis M, Legrand M, Ostermann M et al (2021) Acute kidney injury in the critically ill: an updated review on pathophysiology and management. Intensive Care Med 47:835–850. 10.1007/s00134-021-06454-7 34213593 10.1007/s00134-021-06454-7 PMC8249842

[59] Lee SA, Cozzi M, Bush EL, Rabb H (2018) Distant organ dysfunction in acute kidney injury: a review. Am J Kidney Dis 72:846–856. 10.1053/j.ajkd.2018.03.028 29866457 10.1053/j.ajkd.2018.03.028 PMC6252108

[60] Sato Y, Takahashi M, Yanagita M (2020) Pathophysiology of AKI to CKD progression. Semin Nephrol 40:206–215. 10.1016/j.semnephrol.2020.01.011 32303283 10.1016/j.semnephrol.2020.01.011

[61] Ferenbach DA, Bonventre JV (2015) Mechanisms of maladaptive repair after AKI leading to accelerated kidney ageing and CKD. Nat Rev Nephrol 11:264–276. 10.1038/nrneph.2015.3 25643664 10.1038/nrneph.2015.3 PMC4412815

[62] Sato Y, Yanagita M (2018) Immune cells and inflammation in AKI to CKD progression. Am J Physiol-Ren Physiol 315:F1501-1512. 10.1152/ajprenal.00195.2018 10.1152/ajprenal.00195.2018 30156114

[63] He L, Wei Q, Liu J, Yi M, Liu Y, Liu H et al (2017) AKI on CKD: heightened injury, suppressed repair, and the underlying mechanisms. Kidney Int 92:1071–1083. 10.1016/j.kint.2017.06.030 28890325 10.1016/j.kint.2017.06.030 PMC5683166

[64] Jiang M, Bai M, Lei J, Xie Y, Xu S, Jia Z et al (2020) Mitochondrial dysfunction and the AKI-to-CKD transition. Am J Physiol Ren Physiol 319:F1105-1116. 10.1152/ajprenal.00285.2020 10.1152/ajprenal.00285.2020 33073587

[65] D’Urso A, Brickner JH (2014) Mechanisms of epigenetic memory. Trends Genet 30:230–236. 10.1016/j.tig.2014.04.004 24780085 10.1016/j.tig.2014.04.004 PMC4072033

[66] Wanner N, Bechtel-Walz W (2017) Epigenetics of kidney disease. Cell Tissue Res 369:75–92. 10.1007/s00441-017-2588-x 28286899 10.1007/s00441-017-2588-x

[67] Prachayasittikul V, Prathipati P, Pratiwi R, Phanus-umporn C, Malik AA, Schaduangrat N et al (2017) Exploring the epigenetic drug discovery landscape. Expert Opin Drug Discov 12:345–362. 10.1080/17460441.2017.1295954 28276705 10.1080/17460441.2017.1295954

[68] Tanemoto F, Nangaku M, Mimura I (2022) Epigenetic memory contributing to the pathogenesis of AKI-to-CKD transition. Front Mol Biosci 9:1003227. 10.3389/fmolb.2022.1003227 36213117 10.3389/fmolb.2022.1003227 PMC9532834

[69] Alobaidi R, Anton N, Burkholder S, Garros D, Garcia Guerra G, Ulrich EH, Bagshaw SM (2021) Association between acute kidney injury duration and outcomes in critically ill children. Pediatr Crit Care Med 22:642–650. 10.1097/PCC.0000000000002679 33729733 10.1097/PCC.0000000000002679

[70] Deaton A, Cartwright N (2018) Understanding and misunderstanding randomized controlled trials. Soc Sci Med 210:2–21. 10.1016/j.socscimed.2017.12.005 29331519 10.1016/j.socscimed.2017.12.005 PMC6019115

[71] Rifkin DE, Coca SG, Kalantar-Zadeh K (2012) Does AKI truly lead to CKD? J Am Soc Nephrol 23:979–984. 10.1681/ASN.2011121185 22460531 10.1681/ASN.2011121185 PMC3358766

[72] Goldstein SL, Krallman KA, Roy JP, Collins M, Chima RS, Basu RK, Chawla L, Fei L (2023) Real-time acute kidney injury risk stratification-biomarker directed fluid management improves outcomes in critically ill children and young Adults. Kidney Int Rep 8:2690–2700. 10.1016/j.ekir.2023.09.019 38106571 10.1016/j.ekir.2023.09.019 PMC10719644

[73] Kwiatkowski DM, Menon S, Krawczeski CD, Goldstein SL, Morales DL, Phillips A, Manning PB, Eghtesady P, Wang Y, Nelson DP, Cooper DS (2015) Improved outcomes with peritoneal dialysis catheter placement after cardiopulmonary bypass in infants. J Thorac Cardiovasc Surg 149:230–236. 10.1016/j.jtcvs.2013.11.040 24503323 10.1016/j.jtcvs.2013.11.040

[74] Ryerson LM, Mackie AS, Atallah J, Joffe AR, Rebeyka IM, Ross DB, Adatia I (2015) Prophylactic peritoneal dialysis catheter does not decrease time to achieve a negative fluid balance after the Norwood procedure: a randomized controlled trial. J Thorac Cardiovasc Surg 149:222–228. 10.1016/j.jtcvs.2014.08.011 25218539 10.1016/j.jtcvs.2014.08.011

[75] Ulrich E, Bedi P, Alobaidi R, Morgan CJ, Paulden M, Zappitelli M, Bagshaw S (2024) Outcomes of prophylactic peritoneal dialysis catheter insertion in children undergoing cardiac surgery: a systematic review and meta-analysis. Pediatr Crit Care Med. 10.1097/PCC.0000000000003465 38334438 10.1097/PCC.0000000000003465

[76] Meersch M, Schmidt C, Hoffmeier A, Van Aken H, Wempe C, Gerss J, Zarbock A (2017) Prevention of cardiac surgery-associated AKI by implementing the KDIGO guidelines in high risk patients identified by biomarkers: the PrevAKI randomized controlled trial. Intensive Care Med 43:1551–1561. 10.1007/s00134-016-4670-3. Erratum in: Intensive Care Med. 2017 Mar 7 28110412 10.1007/s00134-016-4670-3 PMC5633630

[77] Göcze I, Jauch D, Götz M, Kennedy P, Jung B, Zeman F, Gnewuch C, Graf BM, Gnann W, Banas B, Bein T, Schlitt HJ, Bergler T (2018) Biomarker-guided intervention to prevent acute kidney injury after major surgery: the prospective randomized BigpAK study. Ann Surg 267:1013–1020. 10.1097/SLA.0000000000002485 28857811 10.1097/SLA.0000000000002485

[78] Höfler M (2005) The Bradford Hill considerations on causality: a counterfactual perspective. Emerg Themes Epidemiol 2:11. 10.1186/1742-7622-2-11 16269083 10.1186/1742-7622-2-11 PMC1291382

[79] Galea S, Riddle M, Kaplan GA (2010) Causal thinking and complex system approaches in epidemiology. Int J Epidemiol 39:97–106. 10.1093/ije/dyp296 19820105 10.1093/ije/dyp296 PMC2912489

