# Supplementary material for: Pediatric acute kidney injury and adverse health outcomes: using a foundational framework to evaluate a causal link
Source: Pediatr Nephrol. 2024 Jul 1;39(12):3425–38. doi: 10.1007/s00467-024-06437-y (PMC11511696; doi:10.1007/s00467-024-06437-y)
Supplement: Supplementary file 1 — Graphical abstract (PPTX 117 KB) [file 467_2024_6437_MOESM1_ESM.pptx]

## Slide 1
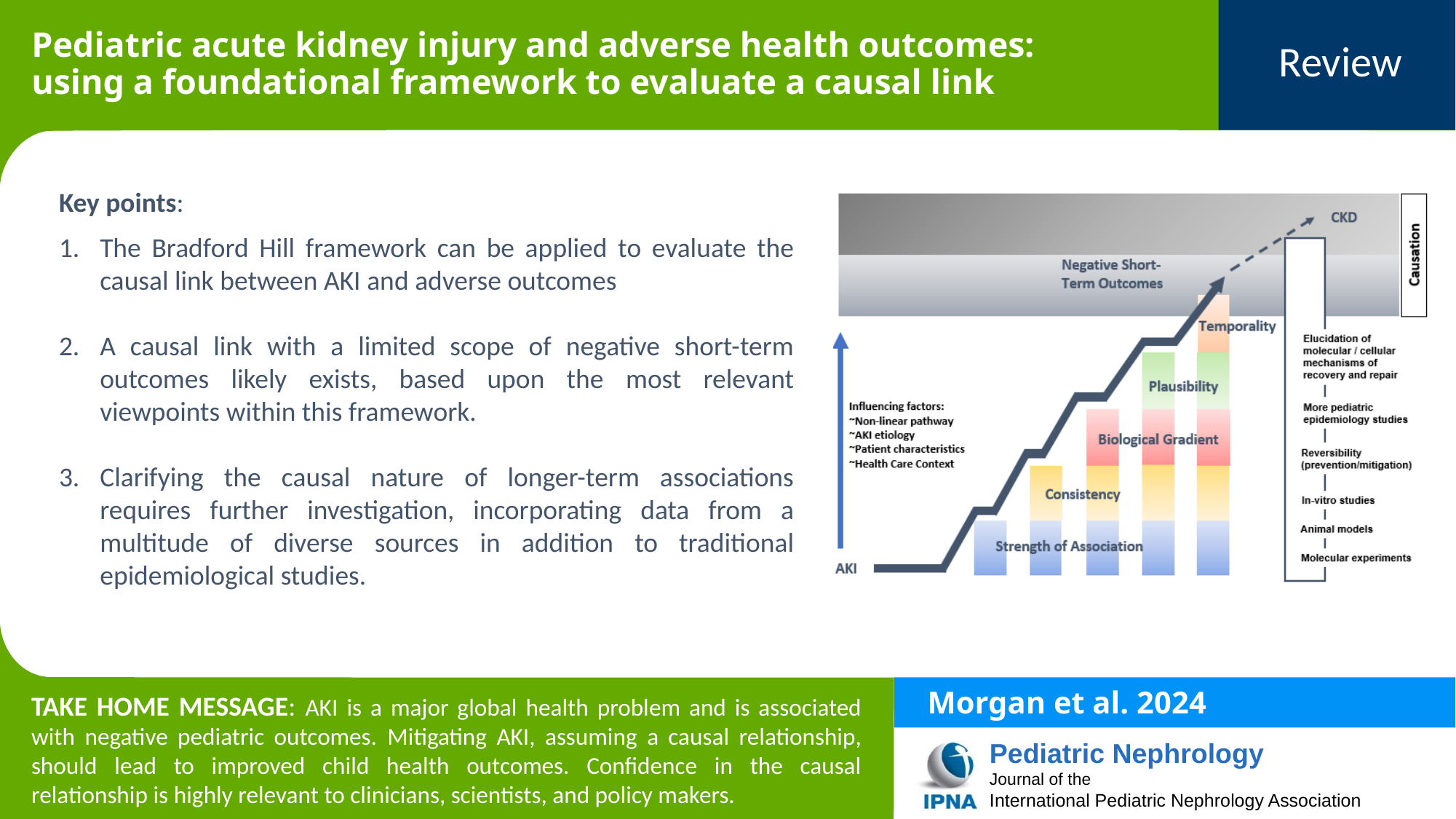

Pediatric acute kidney injury and adverse health outcomes:
using a foundational framework to evaluate a causal link
Key points:
The Bradford Hill framework can be applied to evaluate the causal link between AKI and adverse outcomes
A causal link with a limited scope of negative short-term outcomes likely exists, based upon the most relevant viewpoints within this framework.
Clarifying the causal nature of longer-term associations requires further investigation, incorporating data from a multitude of diverse sources in addition to traditional epidemiological studies.
Morgan et al. 2024
TAKE HOME MESSAGE: AKI is a major global health problem and is associated with negative pediatric outcomes. Mitigating AKI, assuming a causal relationship, should lead to improved child health outcomes. Confidence in the causal relationship is highly relevant to clinicians, scientists, and policy makers.
